# Factors Associated with an Absence of Effect of an Education Program for Improving Knowledge of Schizophrenia

**DOI:** 10.5539/gjhs.v4n4p42

**Published:** 2012-05-22

**Authors:** Hatsumi Yoshii, Yuichiro Watanabe, Hideaki Kitamura, Yoshitaka Sakai, Kouhei Akazawa

**Affiliations:** 1 School of Health Sciences, Faculty of Medicine, Tohoku University, Sendai, Japan; 2 Department of Psychiatry, Niigata University Graduate School of Medical and Dental Sciences, Niigata, Japan; 3 Division of Medical Education, Comprehensive Medical Education Center, School of Medicine, Faculty of Medicine, Niigata University, Niigata, Japan; 4 Department of Medical Informatics and Statistics, Niigata University Graduate School of Medicine, Niigata, Japan

**Keywords:** parents, education program, schizophrenia

## Abstract

Schizophrenia can develop in junior and senior high school students. Correct identification of schizophrenia symptoms is an important factor in subsequent healthcare. The present study conducted a multifaceted evaluation of factors associated with an absence of effect of an education program for improving knowledge of schizophrenia among parents of Japanese junior and senior high school students. Regarding discrimination of prodromal symptoms, the factors associated with an absence of effect of the education program were graduate school education, family income >110 000 USD, proximity to a person with schizophrenia, employment as a professional, and participation in welfare activities for people with mental illness. Regarding discrimination of schizophrenia, the factors associated with an absence of effect were a family income of 53 000 to 110 000 USD (P<0.05), and employment in production/labor service (P<0.05).

## 1. Introduction

Schizophrenia is a serious illness that usually manifests in adolescence or early adulthood (Koning et al., 2009). People with schizophrenia usually experience their first psychotic symptoms before age 20 years (Chong et al., 2004), and the illness can develop in junior and senior high school students. The prognosis for young people is unfavorable (Torrey, 2006), and longer duration of untreated psychosis (DUP) is associated with undesirable outcomes such as delay in remission and an adverse prognosis (Van et al., 2005; Yamazawa et al., 2004; Yung et al., 2007). When left unrecognized, untreated, or poorly treated, psychotic illnesses during adolescence can lead to considerable personal and family distress and increased illness severity. They also contribute to poor academic performance, premature exit from school, unemployment, sustained disability, and early death (Yung et al., 2007). These findings indicate that prompt intervention is critical in ensuring better outcomes. However, several studies have shown an average gap of one or two years between onset of psychosis and beginning of treatment (Boydell et al., 2006). Extensive delays in access to treatment are common after the first episode of a psychotic illness (Lincoln et al., 1995). In general, patients were unaware of their symptoms and reluctant to seek assistance from mental health professionals after their first psychotic episode (Bechard-Evans et al., 2007). Therefore, it is important for parents of junior and senior high school students to be aware of the signs of such episodes in their children. Few studies have assessed whether parents of junior and senior high school students can discriminate symptoms of schizophrenia from symptoms of other physical and psychiatric illnesses. The present authors developed and studied a web-based education program that aimed to improve understanding of schizophrenia among 2465 Japanese parents of junior and senior high school students. This study quantitatively evaluated the factors associated with an absence of effect for this education program on improving parental knowledge of schizophrenia symptoms. The findings are likely to be important in developing education programs for early intervention in schizophrenia.

## 2. Methods

### 2.1 Participants

The participants were selected from an enormous database of 1 370 000 candidates, which was administered by a private Japanese company that specializes in questionnaire research. Stratified random sampling was used to prevent sample bias, and the analysis was stratified by gender and region. Our sample was 2465 parents of junior and senior high school students. All participants completed a questionnaire on an internet website, the details of which have been previously described (Yoshii et al., 2011). Consent from the participants was obtained by the company that administered the database, and the study was approved by the medical ethics committee of Niigata University.

### 2.2 Questionnaire

The questionnaire was used to collect demographic information on the respondents (eg, gender, education) and information on discrimination of symptoms. The questions were based on the *Diagnostic and Statistical Manual of Mental Disorders, Fourth Edition, Text Revision* (DSM-IV-TR) criteria for schizophrenia and on the PRIME-Screen (Kobayashi et al., 2008). The questionnaire comprised 4 items on schizophrenia symptoms, 4 items on prodromal symptoms, and 11 other items. On these scales, higher total scores indicate better discrimination. Parents viewed the education program, and the questionnaire was answered again after 1 week. These are referred to as the “before” and “after” questionnaires. The details of the questionnaire have been previously described (Yoshii et al., 2011).

### 2.3 Web-based Education Program

A web-based education program was developed to improve understanding of schizophrenia. It described the positive and negative symptoms of schizophrenia, as well as the prodromal symptoms of schizophrenia. It comprised 12 narrated slides and required 13 minutes to complete. All parents were invited to view this web-based education program, as previously described (Yoshii et al., 2011).

### 2.4 Statistical Analysis

Analyses were done using the SPSS Version 16.0. Correct answer scores were calculated by dividing the subtract the “before” questionnaire from the “after” questionnaire with those on for all respondents by the number of items. The Student or Welch t-test and one-way analysis of variance were used to identify the education program factors that had no effect. The Kruskal-Wallis test and Mann-Whitney U test were used to examine the associations between the score on the “before” questionnaire and the demographic characteristics of participants. A p value <0.05 was considered to indicate statistical significance, and all statistical tests were two-tailed.

## 3. Results

### 3.1 Characteristics of Participants

The participants were 2465 Japanese parents of junior and senior high school students: 1381 (56.02%) men and 1084 (43.98%) women. Regarding family structure, 1921 (77.93%) household had 2 parents, 82 (3.33%) had 1 parent, and 424 (17.20%) comprised 3 generations. Regarding occupation, 585 (23.73%) were employed in production/labor service and 523 (21.22%) were unemployed. A total of 206 (8.36%) parents had experience participating in mental-health welfare activities (Yoshii et al., 2011).

### 3.2 Factors Associated with an Absence of Effect of the Education Program

Regarding discrimination of schizophrenia, family income (P<0.05) and occupation (P<0.05) were associated with an absence of effect of the education program (Table 1). The lowest mean (±SD) scores for discrimination of schizophrenia were 0.47±1.57 among those with a family income of 53 000 to 110 000 USD and 0.38±1.59 for those employed in production/labor service. Regarding the score on the “before” questionnaire, the results of the Kruskal-Wallis test showed no significant difference between participants categorized by occupation and those categorized by family income (P>0.05; Table 2).

**Table 1:** Factors associated with an absence of effect of the education program

	n	Discrimination of schizophrenia		Discrimination of prodromal symptoms	
		Mean± SD	P*	Mean± SD	P*
**Age (years)**			0.276		0.347
30-39	196	0.36±1.42		1.48±1.95	
40-49	1743	0.55±1.62		1.20±2.04	
50-59	512	0.60±1.65		1.24±2.13	
60-69	14	0.21±2.05		1.21±2.58	
**Gender**			0.937		0.567
Male	1298	0.54±1.61		1.26±2.13	
Female	1167	0.55±1.61		1.21±1.98	
**Education**			0.745		0.001
Junior high school	25	0.80±1.29		1.72±2.17	
High school	690	0.55±1.57		1.53±1.90	
Vocational school	310	0.61±1.64		1.24±2.08	
Junior college	355	0.58±1.65		1.02±1.94	
University	990	0.51±1.62		1.18±2.15	
Graduate school	91	0.46±1.75		0.29±2.08	
Others	4	1.50±2.38		1.00±1.83	
**Family structure**			0.303		0.551
2 parents	1921	0.55±1.63		1.21±2.08	
1 parent	82	0.80±1.67		1.20±1.98	
3 generations	424	0.47±1.53		1.36±1.97	
Others	38	0.76±1.67		1.29±1.99	
**Family income (US dollars)**			0.008		0.002
< 11 000	37	1.00±1.43		1.59±1.98	
11 000–32 000	181	0.81±1.82		1.54±1.87	
32 000–53 000	465	0.67±1.64		1.40±1.99	
53 000–110 000	1326	0.47±1.57		1.22±2.05	
>110 000	456	0.51±1.62		0.95±2.19	
**Proximity to person with schizophrenia**			0.498		0.006
Yes	78	0.42±1.40		0.60±2.25	
No	2387	0.55±1.62		1.25±2.05	
**Occupation**			0.028		0.001
Agriculture, forestry, and fisheries	8	1.75±1.67		1.50±1.51	
Production/labor service	585	0.38±1.59		1.39±2.09	
Transportation and communication	134	0.46±1.58		1.34±2.27	
Sales and marketing	315	0.61±1.57		1.13±2.02	
Service industry	305	0.72±1.73		1.31±2.02	
Professionals	442	0.55±1.57		0.83±2.05	
Others	153	0.53±1.65		1.09±2.01	
Unemployed	523	0.59±1.62		1.42±2.00	
**Participation in welfare activities for people with mental illness**			0.338		0.001
Yes	206	0.44±1.67		0.69±2.21	
No	2259	0.55±1.61		1.28±2.04	

Correct answer scores were calculated by dividing the subtract the “before” questionnaire from the “after” questionnaire with those on for all respondents by the number of items.

* Student or Welch t-test and one-way analysis of variance.

**Table 2:** Scores on “before” questionnaire (factors associated with an absence of effect of the education program)

	**n**	**Discrimination of schizophrenia**		**Discrimination of prodromal symptoms**	
		Maximum score: 4	*P*	Maximum score: 4	*P*
		Median (25%, 75%)		Median (25%, 75%)	
**Education**					0.001
Junior high school	25	-		0.00 (0.00, 2.00)	
High school	690	-		1.00 (0.00, 2.00)	
Vocational school	310	-		1.00 (0.00, 2.00)	
Junior college	355	-		1.00 (0.00, 2.00)	
University	990	-		1.00 (0.00, 2.00)	
Graduate school	91	-		2.00 (1.00, 3.00)	
Others	4	-		1.00 (0.25, 2.50)	
**Occupation**			0.990		0.037
Agriculture, forestry, and fisheries	8	-		0.00 (0.00, 2.00)	
Production/labor service	585	-		1.00 (0.00, 2.00)	
Transportation and communication	134	-		1.00 (0.00, 2.00)	
Sales and marketing	315	-		1.00 (0.00, 2.00)	
Service industry	305	-		1.00 (0.00, 2.00)	
Professionals	442	-		1.00 (0.00, 3.00)	
Others	153	-		1.00 (0.00, 2.00)	
Unemployed	523	-		1.00 (0.00, 2.00)	
**Family income (US dollars)**			0.128		0.001
<11 000	37	-		1.00 (0.00, 2.00)	
11 000–32 000	181	-		1.00 (0.00, 2.00)	
32 000–53 000	465	-		1.00 (0.00, 2.00)	
53 000–110 000	1326	-		1.00 (0.00, 2.00)	
>110 000	456	-		1.00 (0.00, 2.00)	
**Proximity to person with schizophrenia**					0.001
Yes	78	-		2.00 (0.00, 3.00)	
No	2387	-		1.00 (0.00, 2.00)	
**Participation in welfare activities for people with mental illness**					0.001
Yes	206	-		1.00 (0.00, 3.00)	
No	2259	-		1.00 (0.00, 2.00)	

* Kruskal-Wallis test, Mann-Whitney U test

Regarding discrimination of prodromal symptoms, the factors associated with an absence of effect of the education program were education (P<0.05), family income (P<0.05), proximity to a person with schizophrenia (P<0.05), occupation (P<0.05), and participation in welfare activities for people with mental illnesses (P<0.05; Table 1). The lowest mean (±SD) scores for discrimination of prodromal symptoms were 0.29±2.08 for graduate school education, 0.95±2.19 for a family income >110 000 USD, 0.60±2.25 for proximity to a person with schizophrenia, 0.83±2.05 for employment as a professional, and 0.69±2.21 for participation in welfare activities for people with mental illnesses. Regarding the score on the “before” questionnaire, the Kruskal-Wallis test and Mann-Whitney U test showed associations between the dependent variable (base of knowledge) and independent variables (demographic characteristics). It was significantly associated with education, occupation, family income, proximity to a person with schizophrenia, and participation in welfare activities for people with mental illnesses (P<0.05; Table 2).

## 4. Discussion

Schizophrenia tends to have a chronic course and can result in considerable disability. Early intervention might improve both response to antipsychotic treatment and long-term outcome (Yoshii et al., 2012). Awareness of a mental disorder at onset was reported to be related to shorter DUP (Yamazawa et al., 2004). In a series of Australian studies, poor mental-health literacy was a significant barrier to appropriate help-seeking among youths and adults. Because schizophrenia can develop even in junior and senior high school students, a better understanding of the disorder among the parents of adolescents could lead to earlier recognition and intervention (Bechard-Evans et al., 2007; Yung et al., 2007). Thus, developed a schizophrenia education program has been developed for parents of adolescents in Japan.

The present study conducted a multifaceted evaluation of the factors associated with an absence of effect of the education program on improving schizophrenia knowledge among parents of junior and senior high school students. The present study tested the hypothesis that older age would be associated with an absence of effect. The present findings, which confirm those of Farrer, showed that although older adults (≥70 years) were indeed less able than younger age groups to correctly recognize depression and schizophrenia, the lower scores for discrimination of schizophrenia and discrimination of prodromal symptoms did not significantly differ by age. Therefore, age was not associated with the effect of education.

Most people who develop schizophrenia experience a prodromal phase of functioning before the onset of psychotic symptoms. This prodromal phase includes depressed mood, anxiety, irritability, changes in volition, cognitive changes, physical symptoms, behavioral changes, impaired tolerance to normal stress, and attenuated psychotic symptoms. Prodromal symptoms are generally nonspecific (Yoshii et al., 2011). Our educational program improved the ability to distinguish prodromal symptoms (Yoshii et al., 2011). The factors associated with an absence of effect of the education program were those associated with the highest baseline scores for discrimination of prodromal symptoms, ie, graduate school education, family income >110 000 USD, proximity to a person with schizophrenia, employment as a professional, and participation in welfare activities for people with mental illnesses. Knowledge did not increase among people who were already knowledgeable. In other words, the education program had a positive effect among people who did not already know about prodromal symptoms. These results were somewhat disappointing, as it was hoped that the education program would have a broader educational impact.

A low score on a test that measured ability to discriminate schizophrenia was associated with prolongation of DUP (Bechard-Evans et al., 2007; Yung et al., 2007). Therefore, it is important for parents to be able to recognize the symptoms of schizophrenia. Our educational program was able to improve this ability (Yoshii et al., 2011). Therefore, it might be useful in facilitating early intervention. Individuals who reported a family history of psychiatric treatment or a friend with a history of psychiatric treatment had higher knowledge scores than did those without those characteristics (Esterberg et al., 2008). In the present study, the factors associated with an absence of effect of the education program were a family income 53 000 to 110 000 USD and employment in the production/labor service. These factors didn’t the high at base of knowledge.

## 5. Conclusions

In discriminating prodromal symptoms, the factors associated with an absence of effect of the education program were graduate school education, family income >110 000 USD, proximity to a person with schizophrenia, employment as a professional, and participation in welfare activities for people with mental illnesses. In discriminating schizophrenia, the factors associated with an absence of effect were a family income of 53 000 to 110 000 USD and employment in the production/labor service (P<0.05).
